# Investigating the physical stability of repackaged medicines stored into commercially available multicompartment compliance aids (MCAs)

**DOI:** 10.1111/jphs.12176

**Published:** 2017-05-08

**Authors:** Bahijja Tolulope Raimi‐Abraham, Alba Garcia del Valle, Carlota Varon Galcera, Susan Anne Barker, Mine Orlu

**Affiliations:** ^1^ Department of PharmaceuticsSchool of PharmacyUniversity College LondonLondonUK; ^2^ University of BarcelonaBarcelonaSpain; ^3^ King's College LondonInstitute of Pharmaceutical ScienceLondonUK

**Keywords:** compliance, multicompartment compliance aid, older patient, repackaging, stability

## Abstract

**Background:**

Compliance aids are devices which have been developed and are currently used to assist individuals in their medicines management. The use of compliance aids involves the transfer of medicines from the manufacturers' original packaging and repackaged into an multicompartment compliance aid (MCA). MCAs do not guarantee the same level of protection compared to manufacturer's original packaging.

**Objective:**

The aim of this study was to investigate the stability profile of atenolol, aspirin and lansoprazole dosage forms repackaged together in two different commercially available MCAs.

**Methods:**

In a laboratory in the United Kingdom, the physical stability of the formulations repackaged into two commercially available brands of MCAs was evaluated. After 8 weeks of storage (under controlled ambient conditions), changes in the disintegration (tablets only) and dissolution properties (all formulations) were examined in accordance with British Pharmacopoeia (BP) specifications.

**Key findings:**

Findings from this study confirm that changes in solid‐dosage form quality are observed when repackaged into MCAs compared to manufacturers packaging resulting in differences in *in‐vitro* dissolution performance. However, even with these changes, overall product performance was acceptable and within BP specifications.

**Conclusion:**

There is a need for greater collaboration in this area between manufacturers, hospital and community pharmacists, academics and policymakers to increase the data available on the physical stability and in turn performance of medicines repackaged into MCAs.

## Introduction

Compliance aids, also referred to as multicompartment compliance aids (MCAs) or dose administration aids (DAAs), are devices which have been developed and are currently used to assist individuals in their medicines management.^1^ The rationale with most MCAs is that one compartment corresponds to a single administration time‐point and all of the patient's solid‐dose medicines prescribed for that time‐point are dispensed into that compartment.^2^, ^3^, ^4^ Where the frequency of administration does not exceed four times a day, a 28‐compartment MCA provides the patient with a 7‐day dosing regimen for their solid‐dose medication.^2^, ^3^ Participants at a public engagement event in 2013 entitled ‘How to improve medicines for older people?^5^ felt that although MCAs were widely available to improve patient outcomes in terms of medicine management, these aids may actually (in some cases) hinder compliance as oppose to improving it. This could occur due to loss of contextual information on the appropriate use of their medicine when received in an MCA, often leading to an inability to identify which drug is used to treat their specific conditions. Some participants at the public engagement event had experience using automated medication dispensers (which have a self‐locking shutter over the lid preventing direct access to their medication except at the time the device was programmed for them to take their medication). These participants expressed strong concern about not knowing how they would cope if the system broke down.

The stability of a pharmaceutical product can be defined as ‘the capability of a particular medicine, in a specified container, to remain within its physical, chemical, microbiological, therapeutic and toxicological specifications’.^6^ The shelf life of a formulation is greatly affected by the intrinsic stability of the active pharmaceutical ingredient (API), the excipients, the potential interactions between them, the manufacturing process, the packaging and environmental conditions encountered during transport of the product and its storage conditions.^7^ Manufacturers’ packaging is specifically designed to protect the medicinal product from environmental factors encountered during storage, such as light, air (oxygen, carbon dioxide and other gases), and moisture, whilst also limiting the interactions between the dosage form and the packaging material.^7^, ^8^, ^9^ API stability within a dosage form is routinely confirmed according to international regulatory requirements, where stability studies on packaged medicines are conducted under long‐term and accelerated conditions at specific temperatures and relative humidity (RH) to represent storage conditions experienced in the distribution chain of the climatic zone(s) of the country or region of the world concerned.^10^

One of the main roles of manufacturers' packaging is to protect the product from exposure to conditions where phenomena can occur that can alter its performance.^11^ When a solid material has a lower water activity than its surroundings, a process called deliquescence occurs, in which moisture condenses on the solid resulting in a liquid condensate which can dissolve the material.^12^ This process will continue until the water activity of the dissolved material matches that of its surroundings and can result in physical and chemical change (e.g. appearance, dissolution and degradation) of the material.^13^

The use of MCAs involves the transfer of medicines from the manufacturer's original packaging into the MCA. The Royal Pharmaceutical Society (in the United Kingdom) advises that ‘medicines should not be stored in a MCA for longer than 8 weeks’^14^ and have noted (along with Glass and Haywood^7^) that there is a lack of sufficient stability data available to support the repackaging of medicines into MCAs. Anecdotal (from both community and hospital pharmacists) and literature evidence (such as from Haywood *et al*.^15^ and Perks *et al*.^16^) have shown that MCAs are usually given as either a 1‐, 4‐ or 8‐week supply. Results from a survey conducted by Church and Smith^3^ of 392 repackaged drug products revealed that, although some information regarding the potential stability of solid‐dosage forms in MCAs could be obtained from manufacturers, there was still a lack of short‐term stability data for the transfer of drug products into these devices.^3^ Manufacturers, on the whole, discourage the repackaging of medications as there are little data available to support this process.

Only a handful of medications have been investigated for their stability following repackaging into MCAs, namely metoprolol,^17^ aspirin,^18^ atenolol,^2^ clozapine,^16^ furosemide,^19^ paracetamol,^20^, ^21^ prochlorperazine^10^ and sodium valproate.^7^, ^22^

### Aim of the study

The physical stability of dosage forms repackaged into an MCA and performance is an area of importance that has been under‐researched. Investigations into the chemical stability of the dosage forms were outside of the scope of this study and were not conducted. The aim of this study was to investigate the physical stability profile of atenolol, aspirin and lansoprazole dosage forms repackaged together in two different commercially available MCAs.

Formulations of atenolol, aspirin and lansoprazole were investigated due to their physicochemical, stability and therapeutic applications. Atenolol (a beta‐adrenoreceptor antagonist i.e. a beta‐blocker) is used to treat hypertension, angina and some arrhythmias. Atenolol is a light‐sensitive compound and is reported to be photoreactive when exposed to ultraviolet A (UVA) and ultraviolet B (UVB) radiation.^23^ Aspirin (or acetylsalicylic acid) at low doses is prescribed for secondary prevention of thrombotic cerebrovascular and cardiovascular disease and following bypass surgery. Aspirin is hygroscopic and is rapidly hydrolysed to salicylic acid (SA) on exposure to moisture (according to the British Pharmacopoeia (BP), the limit of the SA content within a dispersible aspirin tablets is 3%).^24^ Lansoprazole (a proton pump inhibitor (PPI)) is used for the treatment of duodenal and stomach ulcer, heartburn, acid regurgitation, Zollinger–Ellison syndrome and the treatment or prevention of gastro‐oesophageal reflux disease and duodenal or stomach ulcer caused by Non‐Steroidal Anti‐Inflammatory Drugs (NSAIDs).

### Ethics approval

Ethics approval was not required for this study.

## Methods

### Products

Atenolol 100 mg film‐coated (atenolol FC) tablets, aspirin 75 mg dispersible tablets (aspirin DT) and lansoprazole 30 mg gastro‐resistant capsules (lansoprazole GR‐C) were used in this study and were used as received. Atenolol, aspirin and lansoprazole powders were purchased from Sigma‐Aldrich (Gillingham, UK), Alfa Aesar (Heysham, UK) and Cambridge Bioscience LTD (Cambridge, UK) respectively. Drug powders were used as either purchased or compressed into compacts.

### Sample preparation

An appropriate number of atenolol FC, aspirin DT and lansoprazole GR‐C were repackaged into two commercially available brands of MCAs, referred to as MCA1 and MCA2.

MCA1 consists of a plastic 28‐cell chamber. The individual chambers (~3 × 1.5 × 1.5 cm^3^) are flexible and arranged into seven rows of four in each column covered by an aluminium foil lid.

MCA2 consists of a hard plastic 28‐compartment circular tray. The volume of each compartment is 10 mL. MCA2 is designed to be preprogrammed (up to 24 times daily), and the tray rotates within the dispenser to the next chamber containing the patient's medication which is visible through an aperture in the lid. The alarm signal sounds at preprogrammed time intervals.

Both systems were kept under controlled room temperature conditions (i.e. 20°C/40 %RH) for 8 weeks with the drug powders stored concurrently stored in open Petri dishes under the same conditions. The temperature of a pharmacy must be maintained within a range compatible with the storage of medication (below 25°C). The time frame of 8 weeks was chosen based on policy recommendations, literature and anecdotal evidence.^3^, ^14^, ^15^, ^16^

The physical stability of the repackaged formulations was evaluated at week 0 and after 4 and 8 weeks of storage by examining changes in the water content, disintegration (tablets only) and dissolution properties (all formulations) in accordance with the BP specifications. For the drug powders, water content and changes in the molecular structure, chemical degradation and changes in solid state under conditions were evaluated. This was to observe the changes (if any) to the drug alone without the influence of excipients.

### Tablet morphology

Changes in sample morphology were evaluated using a FEI™ Quanta 200F Field Emission scanning electron microscope (SEM) FEI, (Hillsboro, Oregon, USA) Samples were coated with 20 nm of gold under vacuum using a Quorum Q150T Turbo‐Pumped Sputter Coater Quorum Technologies (Laughton, UK) with a film thickness monitor unit. All micrographs were taken at an acceleration voltage of 5 kV.

### Water content determination using thermogravimetric analysis

The RH inside packaging is assumed to always be in an equilibrium state between its surroundings and the contained product, with moisture transfer into or out of the package being rate‐limited.^11^ The RH within an MCA is expected to be different from that of the original manufacturer package as they are made of different materials.

Water content was determined using thermogravimetric analysis (TGA). Two samples of each formulation were finely ground using a pestle and mortar, and samples were analysed in Perkin Elmer 40 μL 0.15‐mm aluminium pans with an accompanying pinholed lid at 10°C/min over a suitable temperature range using a TA instruments 2950 Hi‐Res TGA (TA instruments, New Castle, DE, USA).

### *In‐vitro* disintegration testing

*In‐vitro* disintegration testing was conducted on atenolol FC and aspirin DT in accordance with the BP specifications (Appendix XII A. Disintegration). Six tablets per storage condition and MCA type were placed separately in the six cylinders of the disintegration apparatus (Pharma Test Dist 3, Hainburg, Germany. Distilled water was used as the medium at 37 ± 0.5°C and 25 ± 0.5°C for atenolol FC and aspirin DT respectively. Disintegration was recorded by the operator as the time‐point corresponding to the breakdown of all six tablets.

### *In‐vitro* dissolution testing

*In‐vitro* dissolution testing was conducted on atenolol FC, aspirin DT and lansoprazole GR‐C according to the BP method (Appendix XII B. Dissolution) using Apparatus 2 (Paddle apparatus). For atenolol FC and aspirin DT, *in‐vitro* dissolution test was conducted in 900 mL of dissolution media (37 ± 0.5°C) at a basket rotational speed of 50 rpm. Ten milliliters sample from each vessel was taken at specified time intervals and replaced with an equal amount of the fresh dissolution media. Six tablets per storage condition per MCA were tested.

BP specifications state that ‘no more than 10% of the API should be dissolved from GR‐C formulations during the acid stage of the dissolution test’. According to the BP, the dissolution methodology for lansoprazole GR‐C involved two steps. In the first step (referred to as the acid stage), formulations were placed in 0.1 M hydrochloric acid (HCl) for 2 h. The second step (referred to as the buffer stage) occurring immediately after the acid stage in the same vessel involved adjusting the pH to pH 6.8 and continuing the *in‐vitro* dissolution test using the same parameters as mentioned above.

To compare the dissolution performances between the different systems in this study, the *f*_2_ equation (Eq. (1)) was used. The *f*_2_ equation is an independent model that measures the similarity in percentage between two profiles of dissolution^25^ usually for bioequivalence studies; *f*_2_ is a logarithmic reciprocal square‐root‐transformation of the square error, which can be expressed by Equation (1)

(1) $$f_{2}=50\times \log\left\{{\left[1+\left(\frac{1}{n}\right)\times \sum_{t=1}^{n}{\left(R_{t}-T_{t}\right)}^{2}\right]}^{-0.5}\times 100\right\},$$

where *n* is the number of time points, *R*_*t*_ is the percentage of drug release of a reference batch at the time *t,* and *T*_t_ is the percentage of drug released at the comparison batch at time *t*. When *f*_2_ is >50 (i.e., 50–100), this indicates the sameness or equivalence of the two compared profiles. Conversely, when *f*_2_ is <50, this suggests that the profiles are different. In this study, the similarity factor was used as a mathematical approach to assess differences in the dissolution profiles acquired.

### Powder X‐ray diffraction

Powder X‐ray diffraction (PXRD) studies of drug powders were conducted on a D/Max‐BR diffractometer (RigaKu, Tokyo Japan) with Cu‐Kα radiation operating at 40 mV and 30 mA over the suitable 2θ range for each API, a step size of 0.02° at 2°/min. Diffractograms produced were analysed using OriginPro 9.0.0.

## Results

### Repackaged formulations

#### Formulation morphology

**Figure 1:**
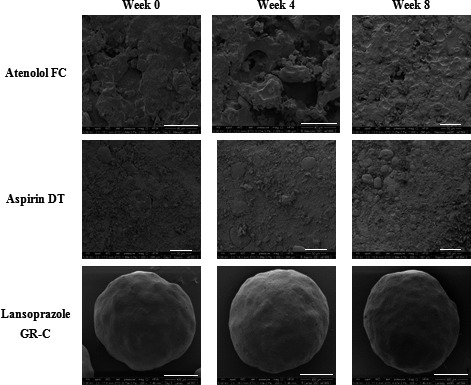
SEM images of atenolol FC, aspirin DT and lansoprazole GR‐C repackaged into the MCA1 stored at ambient conditions. DT, dispersible tablets; FC, film‐coated; GR‐C, gastro‐resistant capsules; MCA, multicompartment compliance aids; SEM, scanning electron microscope.

**Figure 2:**
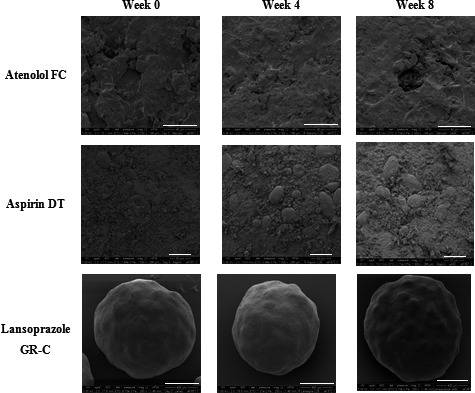
SEM images of atenolol FC, aspirin DT and lansoprazole GR‐C repackaged into the MCA2 stored at ambient conditions. DT, dispersible tablets; FC, film‐coated; GR‐C, gastro‐resistant capsules; MCA, multicompartment compliance aids; SEM, scanning electron microscope.

Figures 1 and 2 show SEM images of the surface of atenolol FC, aspirin DT and lansoprazole GR‐C repackaged into MCA1 and MCA2 stored at ambient conditions for 4 and 8 weeks. No clear changes in morphology were observed in samples stored in either system after 4 and 8 weeks compared to week 0.

#### Water content

TGA studies were conducted to determine the changes in water content of atenolol FC, aspirin DT and lansoprazole GR‐C after repacking into MCA1 and MCA2 and stored under controlled room conditions for 4 and 8 weeks. These results are summarised in Table 1. For atenolol FC samples (stored in both MCA 1 and 2), there was no statistical significant different in values from week 0 to 8. For lansoprazole GR‐C samples stored in both MCAs, a statistically significant difference was observed (p < 0.05, Student's *t*‐test) where a decrease in water content occurred between week 0 and 4 followed by an increase in water content in week 8. Finally, aspirin DT samples saw an increase (p < 0.05, Student's *t*‐test) in water content from week 0 to 8. As aspirin is hygroscopic, it is likely to be susceptible to undergo deliquescence with small variations of RH, running the risk of hydrolysis of aspirin to SA.

**Table 1:** Water content (%) of atenolol FC, aspirin DT and lansoprazole GR‐C at weeks 0, 4 and 8 after repacking into MCA1 and MCA2

	Week 0 (%)	Week 4 (%)		Week 8 (%)	
		MCA 1	MCA 2	MCA 1	MCA 2
Atenolol FC	5.91 ± 0.49	5.72 ± 0.25	5.80 ± 0.08	5.78 ± 0.31	5.85 ± 0.14
Aspirin DT	3.69 ± 0.04	4.43 ± 0.04	4.68 ± 0.13	4.85 ± 0.28	4.80 ± 0.21
Lansoprazole GR‐C	2.36 ± 0.03	1.30 ± 0.04	1.57 ± 0.04	1.41 ± 0.02	1.33 ± 0.04

DT, dispersible tablets; FC, film‐coated; GR‐C, gastro‐resistant capsules; MCA, multicompartment compliance aids.

#### *In‐vitro* disintegration and *in‐vitro* dissolution testing

Overall, faster disintegration times for atenolol FC and aspirin DT were observed after formulations were repackaged into MCA1 and MCA2 for 4 and 8 weeks at room temperature (Table 2). Rapid disintegration and in turn rapid drug dissolution can potentially affect dosage form performance. Common disintegrants are considered to be chemically stable under any ‘normal storage’ conditions but can be greatly affected by moisture uptake.^11^

**Table 2:** Disintegration times for atenolol FC and aspirin DT at weeks 0, 4 and 8 after repacking MCA1 and MCA2

	Week 0 (seconds)	Week 4 (seconds)		Week 8 (seconds)	
		MCA 1	MCA 2	MCA 1	MCA 2
Atenolol FC	154.83 ± 7.78	81.17 ± 3.87	81.83 ± 0.08	104.50 ± 5.36	96.33 ± 0.14
Aspirin DT	45.50 ± 7.40	32.00 ± 5.48	31.5 ± 0.13	40.67 ± 8.36	42.33 ± 0.21

DT, dispersible tablets; FC, film‐coated; MCA, multicompartment compliance aids.

**Figure 3:**
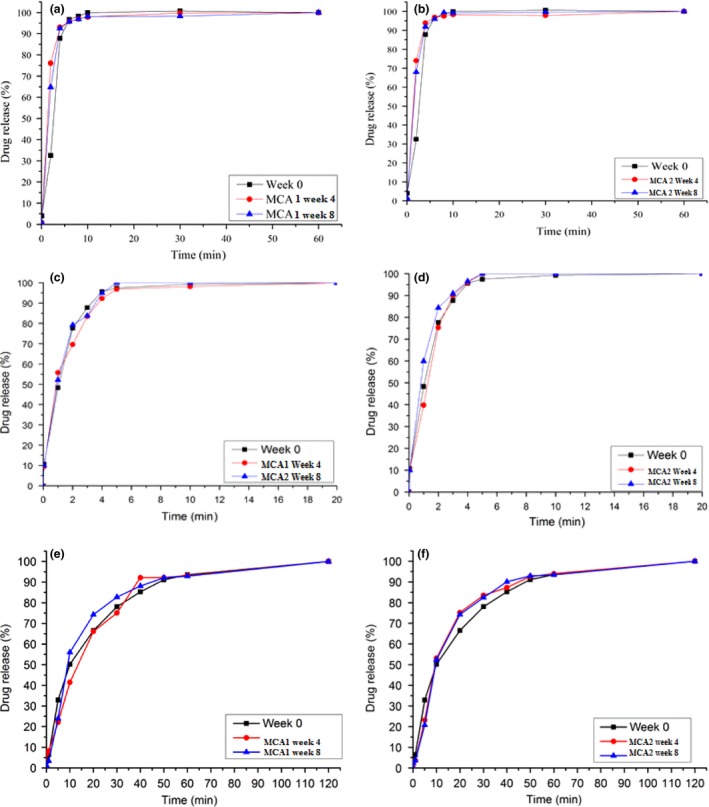
Mean (n = 6) dissolution profiles of original at week 0 (black line) and repackaged in an MCA at week 4 (red line) and 8 (blue line). Atenolol FC repackaged and stored in (a) MCA 1 and (b) MCA 2, aspirin DT repackaged and stored in (c) MCA 1 and (b) MCA 2, lansoprazole GR C repackaged and stored in (e) MCA 1 and (f) MCA 2. Standard deviation bars are not displayed because they are smaller than the symbol. DT, dispersible tablets, FC, film‐coated, GR‐C, gastroresistant capsules; MCA multicompartment compliance aids.

British Pharmacopoeia dissolution criteria states that for conventional‐release formulations, at least 80% of the API should be released within 45 min. Both atenolol FC and aspirin DT formulations (Figure 3a–d) repackaged and stored under ambient conditions in both MCA1 and MCA2 passed according to this criteria. However, a faster dissolution was observed in both formulations (in both MCA1 and MCA2) compared to that observed at week 0.

The dissolution methodology for lansoprazole GR‐C involves two steps in accordance with BP 2013, the acid step and buffer step. The criteria state that in the acid step, no more than 10% of the API should be dissolved from the GR‐C formulation. For repackaged lansoprazole GR‐C stored in MCA1, after 4 and 8 weeks storage under ambient conditions, ~13% and ~10% of API was dissolved in 2 h, compared with ~5% at week 0 (p < 0.05, Student's *t*‐test). . Whilst for repackaged lansoprazole GR‐C stored in MCA2 after 4 and 8 weeks storage, <10% of the API was dissolved in 2 h. In the buffer stage, all lansoprazole GR‐C met the BP dissolution criteria for GR‐C of at least 80% drug released in 45 min. Data showed more drugs was released in the first 5 min from repackaged lansoprazole GR‐C stored in both MCA1 and MCA2 for 4 and 8 weeks (Figure 3e,f) at 22.08% and 25.00 % compared to 7.71% at week 0.

In this study, the *f*_2_ equation was used as a mathematical approach to assess differences in the dissolution profiles acquired. The similarity factors (f2) for the dissolution profiles of the formulations after repackaging into MCA1 and storage for 8 weeks compared to the time 0 dissolution profiles were 45, 79 and 65% for atenolol FC, aspirin DT and lansoprazole GR‐C, respectively. For MCA2, the equivalent values were 43, 63 and 63%. These results suggest that the dissolution profiles of atenolol FT change after repackaging into either MCA1 or MCA2 and subsequent storage for 8 weeks, whereas the dissolution profiles of both aspirin DT and lansoprazole GR‐C are still comparable with the time 0 profiles.

### Active pharmaceutical ingredient powders

#### Formulation morphology

**Figure 4:**
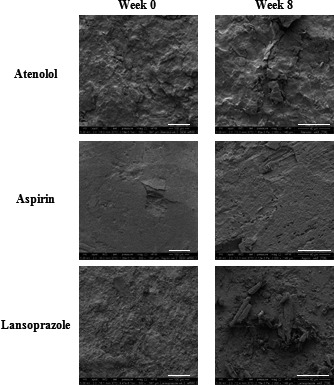
Scanning electron microscope images of atenolol, aspirin and lansoprazole powders at week 0 and after 8 weeks stored at ambient conditions.

Figure 4 shows SEM images of atenolol, aspirin and lansoprazole powders stored at ambient conditions. No clear changes in morphology were observed in the samples at week 8 compared with week 0.

#### Water content

Unlike repackaged (in both MCA1 and MCA2) atenolol FC and lansoprazole GR‐C dosage forms which saw a general decrease in water content over 8‐week storage, atenolol and lansoprazole powders saw an increase in water content after 8 weeks of storage (Table 3).

**Table 3:** Water content (%) of atenolol, aspirin and lansoprazole powders after weeks 0 and 8 storage in ambient conditions

	Water content (%)	
	Week 0	Week 8
Atenolol	2.33 ± 0.34	4.06 ± 0.55
Aspirin	2.02 ± 0.14	2.46 ± 0.07
Lansoprazole	1.46 ± 0.17	2.90 ± 0.34

#### Powder X‐ray diffraction studies

**Figure 5:**
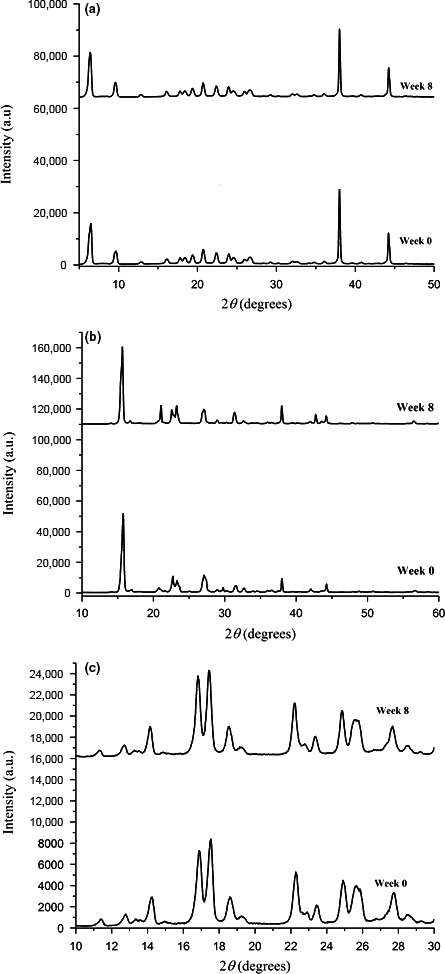
Powder X‐ray diffraction (PXRD) diffraction pattern of repackaged (a) atenolol powders stored under ambient conditions for 8 weeks. (b) PXRD diffraction pattern of repackaged aspirin powders stored under ambient conditions for 8 weeks. (c) PXRD diffraction pattern of repackaged lansoprazole powders stored under ambient conditions for 8 weeks.

Figure 5 shows the PXRD diffraction patterns of repackaged atenolol, aspirin and lansoprazole powders stored under ambient conditions for 8 weeks. Changes in PXRD diffraction peak positions and intensities were observed in aspirin powders, suggestive of changes possibly associated in bond arrangement or solid state (i.e. due to hydrolysis of aspirin to SA), from week 0 and 8.

## Discussion

The physical stability profile (in accordance with the BP specification) of atenolol FC, aspirin DT and lansoprazole GR‐C repackaged together was investigated in two different commercially available and most commonly used MCAs and stored for 8 weeks (under controlled room conditions). The physical stability of drug powders was also stored (under the same conditions) for 8 weeks to observe the changes (if any) to the drug alone without the influence of excipients.

No statistically significant differences in water content were observed for repackaged atenolol FC after 8 weeks of storage; however, atenolol drug powders saw a significant increase in water content. For aspirin DT and lansoprazole GR‐C, statistically significant differences were observed in water content (i.e. increase for aspirin DT and decrease for lansoprazole GR‐C) with little change observed for corresponding drug powders alone. These findings suggest that the presence of excipients (including film coat and capsule shell) may influence the water uptake mechanisms within a formulation, which in turn could have an impact on the dosage form properties and performance. The *in‐vitro* disintegration and dissolution properties of repackaged formulations were faster compared to week 0. Interestingly, the rate of lansoprazole release from GR‐C showed a different profile in the acid stage with more of the drug being released in weeks 4 and 8 compared to week 0. This resulted in more drug being released in the early *in‐vitro* dissolution in buffer stage; however, overall irrespective of MCA type, lansoprazole dosage forms at weeks 4 and 8 met BP dissolution criteria (i.e. at least 80% of drug released in 45 min).

Findings from this study confirm that changes in solid‐dosage form quality are observed when repackaged into MCAs compared to manufacturers' packaging resulting in differences in *in‐vitro* dissolution performance. Although even with these changes, overall product performance was acceptable, and within BP specifications, the chemical stability should be further investigated via thermal analysis methods. Manufacturers' generally discourage the repackaging of medicines due to insufficient data available to support this process; however, the use of MCAs is common practice in healthcare settings especially community. Repackaging of a medication invalidates the stability guarantee of the manufacturer, and in most cases, the responsibility to prepare MCAs falls to nursing staff, pharmacists or pharmacy staff.^1^, ^15^

There is a need for greater collaboration in this area between manufacturers, hospital and community pharmacists, academics and policymakers to increase the data available so that more informed decisions can be made by the healthcare team for the patients taking into account the benefit–risk associated with repackaging of medicines into MCAs.

## Conclusion

Overall, this study confirmed that changes in atenolol, lansoprazole and aspirin dosage form quality occurred when repackaged into MCAs resulting in differences in *in‐vitro* dissolution performance. Even with these changes however, product performance was deemed acceptable and within BP specifications. Due to the lack of sufficient data available to support the repackaging of medicines into MCAs, there is a greater need for cross‐disciplinary collaborations to increase data available to ensure informed decisions can be made by the healthcare team for patients which take into account the benefit–risk associated with MCAs.

## Declarations

### Conflict of interests

The Author(s) declare(s) that they have no conflicts of interest to disclose.

### Funding

This research received no specific grant from any funding agency in the public, commercial or not‐for‐profit sectors.

### Authors’ contributions

All Authors state that they had complete access to the study data that support the publication.
